# Sozoiodol a Substitute for Iodoform

**Published:** 1888-11

**Authors:** 


Sozoiodol a Substitute for Iodoform.—Sozoiodol is a derivative of the aromatic series, and is a phenol, each of whose atoms of hydrogen is replaced by the radical ($SO^3H$), and an atom of iodine. An efficaciousness equal to that of iodoform is attributed to it for the treatment of skin diseases, and it is also said to be (*Rundschau*, No. 50,) equal to salicylic acid in these troubles. The product appears in fine, brilliant crystals; is inodorous; dissolves with difficulty in cold water and cold alcohol; but dissolves much more readily in both substances aided by heat. Sozoiodol contains forty-two per cent. of iodine; it is recommended as a substitute for iodoform, on account of its freedom from odor.—*L' Union pharm.*

